# Reuse of vegetable wastes in animal feed: the influence of red beet powder supplementation on performance, egg quality, and antioxidant capacity of layer quails

**DOI:** 10.1007/s11250-023-03556-w

**Published:** 2023-04-06

**Authors:** Ainhoa Sarmiento-García, Osman Olgun, Gözde Kilinç, Behlül Sevim, Seyit Ahmet Gökmen

**Affiliations:** 1 Área de Producción Animal, Departamento de Construcción Y Agronomía, Facultad de Ciencias Agrarias Y Ambientales, Universidad de Salamanca, 37007 Salamanca, Spain; 2 Department of Animal Science, Faculty of Agriculture, Selcuk University, 42130 Selcuklu, Konya, Türkiye; 3 Department of Food Processing, Suluova Vocational Schools, Amasya University, 05500 Amasya, Türkiye; 4 Eskil Vocational School, Aksaray University, 68800 Aksaray, Türkiye

**Keywords:** Antioxidant, Circular economy, Egg quality, Performance, Quail, Red beet

## Abstract

The survey was carried out to establish the impact of red beet powder (RBP) on performance parameters and egg quality in laying quails. One twenty hundred (120) female laying quails aged 22 weeks were randomly assigned into five groups of 4 females each, and six replicates. Treatments diets were formed by adding 0, 0.2, 0.4, 0.6, and 0.8% RBP to the basal diet. The dietary inclusion of RBP did not affect performance parameters and egg production (*P* > 0.05) except feed conversion ratio, which was quadratically affected (*P* < 0.05). The feed conversion ratio was improved in the 0.4% group with concerning control. The inclusion of RBP did not modify the yolk color (L*, a*, b*) or egg quality (*P* > 0.05). However, the yolk index showed the highest value (*P* < 0.05) in quails fed 0.2% RBP. Free radical scavenging capacity (DPPH) of the yolk decreased (*P* < 0.05) when RBP levels increased above 0.6%. In contrast, the 0.6% RBP group had the highest level of thiobarbituric acid reactive substances (TBARS). Data from the present study provide valuable information to include RBP as an ingredient without affecting performance and egg production. It is an interesting option within the framework of the circular economy and of reusing vegetable products to use this ingredient in animal feed.

## Introduction

An estimated 1.3 billion tons of world food are wasted or lost, which accounts for one-third of all food produced (Georganas et al., 2020). The agricultural industry is concerned about the wastes and by-products resulting from processing fruits and vegetables. There are considerable environmental impacts and economic losses associated with these wastes (Domínguez et al., 2020). While reducing food waste should be a key objective, the re-introduction of food by-products into the food supply chain is essential to implementing the circular economy (Georganas et al., 2020; Sabater et al., 2020). As defined by the bioeconomy, the circular economy uses renewable biological resources to generate value-added products such as food and animal feed (Georganas et al., 2020; Mak et al., 2020). Food waste can be considered a potential ingredient due to its nutritional composition (Espro et al., 2021), which impacts animal feed production (Sabater et al., 2020). For the food and meat industries, food waste provides an economical alternative source of valuable compounds (Domínguez et al., 2020).

Several bioactive compounds are found in residues from vegetable processing (Costa et al., 2017). A bioactive compound is a substance in food that can benefit the body but is present in small amounts. Therefore, their inclusion in animal diets offers opportunities to develop value-added products (Truong et al., 2019). In this context, researchers have focused on using this waste to develop new food products and ingredients (Costa et al., 2017). Beetroot, beetroot juices, powders, extracts, and any other processing residues provide an opportunity to consider them as functional ingredients (Domínguez et al., 2020).

The beetroot plant (*Beta vulgaris*) belongs to the *Chenopodiaceae* family and contains many species, from yellow to red. Due to their wide consumption, dark red roots are particularly popular (Şengül, 2021). The red beet (*Beta vulgaris rubra*) contains a wide range of bioactive phytochemicals, including betalain pigments, betaxanthin, carotenoids, polyphenols, and flavonoids (Marrone et al., 2021). These compounds have antioxidant, anti-ischemic, anti-inflammatory, anti-cancerogenic, and hepatoprotective properties (Chhikara et al., 2019). Moreover, the consumption of red beets has been linked to combating diabetes, lowering blood pressure, preventing heart disease, inhibiting lipid peroxidation, strengthening the immune system, facilitating digestion, and regulating intestinal activity (Şengül, 2021). Moreover, due to the high content of bioactive compounds, the use of red beet as animal feed offers an interesting way to enhance meat shelf life (Domínguez et al., 2020).

Beetroot waste could be an interesting option to use as an ingredient in animal feed contributing to the circular economy. Changes in animals’ feeding are the most commonly studied and applied way to modify meat properties and quality (Vieira et al., 2021; Gül et al., 2022). Earlier authors suggest that, due to the composition of red beets, their inclusion in poultry diets could modify productive performance and affect certain egg parameters, such as yolk color or antioxidant compounds (Kopřiva et al., 2014; Souza et al., 2019; Al-Waeli et al., 2021; Şengül, 2021). However, few researchers have investigated beetroot as animal feed, and the majority of recent work has focused on the application of beetroot as an additive in meat preparations (Domínguez et al., 2020). Moreover, only one study examines the effects of adding RBP to quails’ diets (Şengül, 2021). The Japanese quail is a useful species for avian research, and their production for egg and meat consumption has increased significantly in recent years (Sarmiento-García et al., 2022). The objective of the study was to determine the effects of feeding graded levels of red beet powder on laying quails’ performance and egg quality.

## Materials and methods

### Animals and feed materials

The experiment was performed in an indoor local farm at Selçuklu, Konya, Türkiye (38° 1′ 36″, 32° 30′ 45″). The experiment lasted 10 weeks, between April and June 2022. A total of one hundred twenty (120) laying females quails (*Coturnix coturnix Japonica*) of approximately the same body weight (259.30 ± 5.37 g) and 22 weeks of age were utilized in the research.

Quails were randomized to five treatment groups corresponding to experimental diets and six replicates per treatment, each containing four female quails. Clean, sanitized battery cages (30 cm wide and 45 cm long), a well-ventilated room with the same environmental conditions (20 ± 2.0 °C), and a lighting program of 16 h were used to house the quails.

The five experimental treatments included graded RBP levels in the feed mixture. A total of five experimental diets used in the experiment were formed with the addition of 0, 0.2, 0.4, 0.6, and 0.8% RBP to the diet. RBP was obtained from vegetable waste from a local market (Dağinciri Ltd. Şti., Aydın, Türkiye). The experimental groups received isoproteic and isoenergetic diets. The basal diet presented in mash form was designed following the National Research Council (1994) recommendations to meet the nutritional requirements of layer quail.

As described in AOAC (2006), the chemical composition of the basal diet was determined by incineration and drying the water to determine the ashes (942.05), measuring the protein and fat content by Kjeldahl (990.03) and Soxhlet methods (2003.06), and analyzing the moisture content (2001.12) by drying at 105 °C. Feed and water were supplied ad libitum throughout the 70-day experimental period. Table 1 demonstrates the chemical composition and the ingredients of the basal diet.

**Table 1:** Ingredients and chemical composition of basal diet

Ingredients	%	Chemical composition	%
Corn	57.00	Metabolizable energy (kcal ME/kg)	2898.410
Soybean meal (46% CP)	26.10	Dry matter	87.37
Full-fat soybean	6.50	Crude protein	20.040
Meat-bone meal (45% CP)	2.76	Crude fiber	2.830
Sunflower oil	1.71	Crude fat	5.330
Limestone	5.20	Moisture	12.630
Salt	0.30	Lysine	0.994
Premix^1^	0.25	Methionine	0.449
DL-methionine	0.18	Cystine	0.410
		Calcium	2.500
Total	100.00	Total phosphorus	0.620
		Available phosphorus	0.350

### Determination of performance parameters

Upon arrival at the facility, the quails were weighed and randomly distributed according to the experimental diets. To determine body weight changes, the quails were weighed (± 0.01 g) both at the start and the close of the trial, and the difference was calculated. Each subgroup received experimental diets by weighing each subgroup, and feed intake was calculated by subtracting the quantity supplied from the leftovers from each experimental unit as shown by Souza et al. (2019). The calculated result was divided by the number of quails and by the days the quails fed. The feed intake is expressed in g/bird/day. Average body weight gain (g/day) was determined by subtracting initial body weight (g) from final body weight (g) over the study period.

Eggs were collected at the same time (at 10:00 am) on each day of the experiment. To determine egg production, the number of eggs recorded per day was divided by the number of birds and multiplied by 100. The value was given as a percentage (%).

In order to determine the egg weight, each egg collected during the last 3 days of the research was weighed with a precision weighing balance (± 0.01 g). Concerning these values, egg mass was determined according to Eq. (1):

1 $$Egg\;mass= \frac{\left(egg\;production\;\left(\%\right)\times\;egg\;weight\;\left(g\right)\right)}{100}$$

Lastly, the feed conversion ratio was determined following Eq. (2):

2 $$Feed\;conversion\;ratio=\;\frac{feed\;intake\;(g\;feed)\;}{egg\;mass\;(g\;egg)}$$

### Determination of egg quality parameters

All the procedures described below were determined at the Egg Quality Laboratory (Faculty of Agriculture, Selcuk University, Konya, Türkiye). From all eggs obtained in the last 3 days of the experiment, the internal and external quality parameters were analyzed at room temperature.

Cracked, broken, and damaged eggs throughout the experiment were registered and it was established as a percentage of the whole egg total. A cantilever system is used to determine breaking strength by applying a growing pressure on the wide pole of the shell using the Egg Force Reader. (Orka Food Technology Ltd., Ramat Hasharon, Israel). The three sections (equator, blunt, and pointed parts) values of the eggshells were determined using a digital micrometer (Mitutoyo, 0,01 mm, Japan). These values were used to establish eggshell thickness (µm).

To assess internal egg quality, the eggs were cracked on a cleaned glass surface, and the residues in the eggshell were removed. To determine the eggshell weight, they were previously air-dried at room temperature for 3 days. The weight of the shell was estimated as a ratio to the egg’s weight. Subsequently, the albumen was removed from the yolk. Albumen and yolk heights were determined with a height gauge, and length and width with a 0.01 mm digital caliper (Mitutoyo, Japan). The above values were used to determine the albumen index (3):

3 $$Albumen\;index=\frac{Albumen\;height\;(mm)}{\frac{Albumen\;width+Albumen\;length}{2}\;(mm)}\;x\;100$$

The yolk index was determined according to Eq. (4):

4 $$Yolk\;index=\frac{Height\;of\;yolk}{Diameter\;of\;yolk\;(mm)}\;x\;100.$$

Lastly, the Haugh unit was obtained from the egg weight and albumen height data in accordance with Eq. (5) provided by Stadelman and Cotterill (1995).

5 $$Haugh\;unit\;=\;100\;x\;\mathrm{log}\;(albumen\;height\;+\;7.57\;-\;1.7\;x\;{EW}^{0.37})$$

For colorimetric analysis, the samples were placed in Petri dishes which allowed for maintaining the integrity of the egg yolks. To determine L* (lightness), a* (redness), and b* (yellowness) values, egg yolks were tested with a pre-calibrated Konica Minolta digital colorimeter (Minolta Chroma Meter CR 400 (Minolta Co., Osaka, Japan) as described Titcomb et al. (2019).

### Determination of TBARS and DPPH concentrations in the yolk

To determine lipid peroxidation, the modified TBARS assay proposed by Kilic and Richards (2003) and Sarmiento-García et al. (2021) was performed three times for each sample. Two grams of the yolk was collected and mixed with 12 mL of the trichloroacetic acid (TCA) solution. The solution was blended for 20 s in ULTRA-TURRAX (IKA, USA) and it was filtrated. The mixture was poured into tubes, and 3 mL of the thiobarbituric acid (TBA) solution (0.02 M) was incorporated. The mixture was boiled in a water bath for 40 min to acquire a pink color and centrifuged for 5 min at 2000 rpm. The supernatant was spectrophotometrically determined at a 530 nm wavelength in a spectrophotometer (PerkinElmer, USA) versus a blank consisting of 1 mL TCA extraction solution and 1 mL TBA solution. The TBARS were estimated from a standard curve of malondialdehyde, used for preparing the reference curve. TBA (6) was determined as µmol MDA/g yolk according to Eq. (6):

6 $$TBA\;Value=\frac{(absorbance/k\;x\;2/1000)\;x\;0.8\;}{sample\;weight}\;X\;100$$

The antioxidant capacity of hydrolysates was tested on the radical scavenging effect 1, 1-diphenyl-2-picrylhydrazyl (DPPH)-free radical activity following the adapted method proposed by Sacchetti et al. (2005). Two grams of yolk was isolated and diluted in 25 mL of 95% methanol, then the removal procedure was carried out in an ultrasonic bath for 20 min. The mixture was filtrated and collected in 0.1-mL glass tubes. 2.9 mL of DPPH solution (100 mL of methanol (100%) + 0.0025 g of DPPH (97%)) was incorporated into the solution, then it was mixed for 25 s in a vortex. Once the mix has been allowed to stand at temperature for 30 min, the absorbance was measured using a spectrophotometer (PerkinElmer precisely UV/VIS Spectrometer) at 517 nm wavelength. Control was performed similarly, with 95% ethanol replacing the sample solution. To determine the average value, each experiment was carried out in triplicate. Equation (7) described by Sacchetti et al. (2005) was used to calculate DPPH values:

7 $$DPPH\;values\;=\;\frac{[(Control\;absorbance\;-\;Sample\;absorbance)\;}{Control\;Absorbance}\;x\;100$$

### Statistical analysis

Results were statistically evaluated for each parameter tested. All available data were examined by one-way ANOVA with the SPSS 22.0 software package (SPSS Inc., Chicago, IL, USA). A *p*-value less than 0.05 was taken as statistically significant, while a *p*-value less than 0.10 was set as a tendency. Orthogonal polynomial contrasts for determining the significance of linear and quadratic models were used. It was tested to describe the response of the variable to an increasingly high level of red beet powder.

## Results

### Performance parameters

In all experimental groups, no mortality or symptoms of the disease were observed at the end of the trial. The findings in Table 2 indicated that feeding different levels of RBP did not affect (*P* > 0.05) performance parameters of laying quails (in terms of initial body weight, final body weight, body weight change, and feed intake). Similarly, there were no significant differences (*P* > 0.05) in egg production (including egg weight or egg mass). For the feed conversion rate (FCR), a trend was found (*P* < 0.1). The FCR of the experimental groups fed with diets containing 0.4% RBP was lower than the control group (2.67 vs. 2.97). Moreover, a quadratic trend was observed for egg weight (*P* = 0.080) and egg mass values (*P* = 0.051). In both cases, the highest values of these parameters were observed when quails received 0.4% RBP in the diet.

**Table 2:** Effect of addition of red beet powder in the diet on performance parameters and egg production of laying quails

Parameters	Red beet powder (%)					S. E. M*	P	L	Q
	0	0.2	0.4	0.6	0.8				
Initial body weight (g)	255.5	256.8	266.2	256.3	255.2	5.13	0.536	0.940	0.224
Final body weight (g)	272.3	270.3	279.3	274.7	274.3	5.13	0.844	0.645	0.634
Body weight gain (g)	16.83	13.50	13.08	18.42	19.17	2.54	0.425	0.287	0.194
Feed intake (g/quail/day)	34.67	34.50	33.47	33.28	33.28	0.818	0.631	0.149	0.720
Egg production (%)	90.53	91.21	92.43	92.51	91.86	0.781	0.450	0.158	0.249
Egg weight (g)	12.94	12.96	13.62	12.97	12.41	0.337	0.261	0.366	0.080
Egg mass (g/quail/day)	11.73	11.83	12.58	11.99	11.40	0.337	0.232	0.662	0.051
FCR (g feed/g egg)	2.97	2.92	2.67	2.79	2.92	0.078	0.087	0.352	0.024

*FCR* feed conversion ratio, S. E. M.* standard error means, *L* linear effect, *Q*, quadratic effect

### Egg quality parameters

According to Table 3, the minimum and maximum values of the external parameters of the eggs were as follows: damaged egg (0–0.55%), egg-breaking strength (1.43–1.57 kg) eggshell weight (8.04–8.47%), and eggshell thickness (240.6–245.1 µm**)**. However, no significant differences were detected in damaged eggs, egg-breaking strength eggshell weight, and eggshell thickness among all dietary treatments.

**Table 3:** Effect of addition of red beet powder in the diet on egg external quality in laying quails

Parameters	Red beet powder (%)					S. E. M*	P	L	Q
	0	0.2	0.4	0.6	0.8				
Damaged egg (%)	0.55	0.26	0.00	0.43	0.27	0.225	0.649	0.635	0.341
Egg-breaking strength (kg)	1.43	1.55	1.49	1.54	1.57	0.072	0.718	0.282	0.818
Eggshell weight (%)	8.04	8.48	8.06	8.33	8.47	0.145	0.116	0.143	0.871
Eggshell thickness (µm)	240.6	244.4	245.1	243.4	243.3	4.20	0.960	0.760	0.539

S. E. M.* standard error means, *L* linear effect, *Q* quadratic effect

As can be observed in Table 4, no differences (*P* > 0.05) were observed among treatments for the albumen index and Haugh unit, while the yolk index was affected by RBP diets (*P* < 0.05). The use of 0.2% RBP in laying quail diets significantly increased the yolk index compared to the rest of the studied groups. The lowest value in terms of yellowness was observed in the control group, while the highest value was recorded in the 4 g/kg RBP-supplemented group.

**Table 4:** Effect of addition of red beet powder in the diet on egg internal quality in laying quails

Parameters	Red beet powder (%)					S. E. M*	P	L	Q
	0	0.2	0.4	0.6	0.8				
Albumen index	2.38	2.53	2.36	2.10	2.59	0.142	0.181	0.996	0.289
Yolk index	45.37^b^	48.27^a^	45.36^b^	44.71^b^	45.84^b^	0.749	0.037	0.308	0.668
Haugh unit	86.14	88.52	87.02	83.75	88.95	1.192	0.053	0.834	0.420
L*	51.11	51.74	51.21	51.24	49.75	0.816	0.523	0.227	0.243
a*	1.27	0.75	1.21	0.65	2.21	0.688	0.564	0.435	0.250
b*	33.00	34.77	35.03	33.12	33.68	0.557	0.097	0.879	0.063

L* lightness, a* redness, b* yellowness, S. E. M.* standard error means, *L* linear effect, *Q* quadratic effect. ^a,b^Means with different superscripts in the same row were significantly different (*P* < 0.05)

The colorimetric parameters of the yolk showed similar values in all the groups studied (*P* > 0.05). Those values ranged between 49.75 and 51.74 for lightness (L*), 0.65 to 2.21 for redness (a*), and 33.0 to 35.03 for yellowness (b*). Nevertheless, a trend (*P* = 0.097) was observed for the yellowness parameter. Samples from the control group were less yellow than those from the group that had received 0.4% RBP in the diet.

### TBARS and DPPH concentrations in the yolk

There were significant differences in TBARS (*P* < 0.05) and DPPH (*P* < 0.01) concentrations of yolk between dietary treatments (Table 5). Regarding yolk TBARS levels, those values increased linearly from the control group to the group that received 0.6% RBP in the diet (4.48 to 5.82 µmol MDA/kg). However, the inclusion of 0.8% RBP in the diet decreased TBARS value to levels similar to those obtained in the control group (3.84 µmol MDA/kg).

**Table 5:** Effect of addition of red beet powder in the diet on TBARs and DPPH concentrations of yolk in laying quails

Parameters	Red beet powder (%)					S. E. M*	P	L	Q
	0	0.2	0.4	0.6	0.8				
TBARS (µmol MDA/kg)	4.48^b^	4.94^ab^	5.02^ab^	5.82^a^	3.84^b^	0.407	0.023	0.759	0.010
DPPH (%)	9.46^a^	9.55^a^	9.24^a^	9.74^a^	7.70^b^	0.381	0.006	0.013	0.029

*TBARS* thiobarbituric acid reactive substances, *MDA* malondialdehyde, *DPPH* 2,2 diphenyl-1-picrylhydrazyl, S. E. M.* standard error means, *L* linear effect, *Q* quadratic effect. ^a,b^Means with different superscripts in the same row were significantly different (*P* < 0.05)

Free radical scavenging capacity was estimated as DPPH reduction to determine antioxidant activity spectrophotometrically. Despite what has been observed for TBARS concentration, the lowest value observed for DPPH was at 0.8% RBP diet. The rest of the groups studied, including the control group, showed similar DPPH values.

## Discussion

### Performance

Wastes and by-products, in the form of pods, peels, pulp, and seeds, generated during the processing of fruits and vegetables are an important issue to the agricultural industry. Accordingly, special attention has been paid to reuse food waste to be used to formulate animal feed (Domínguez et al., 2020). Including RBP as an ingredient in animal feed has great advantages due to its high production, low price, and good quality compounds (Costa et al., 2017; Chhikara et al., 2019; Domínguez et al., 2020). The inclusion of RBP in diets did not negatively affect laying quails’ performance or egg production. Likewise, in research determining the impact of the inclusion of 0.8% of beetroot or carrot meal on the performance parameters of laying hens, no differences were found (Souza et al., 2019), which is consistent with the findings of Şengül (2021) in quails. However, laying quails fed a diet containing 0.4% RBP had a better feed conversion ratio value. However, the poorest values were reported for the laying quails fed the control diet. Beetroot compounds may influence digestion efficiency (Chhikara et al., 2019) through the presence of carbohydrates, primarily pectin (de Oliveira et al., 2021). The metabolism of carbohydrates from beet by intestinal bacteria has been demonstrated to modulate gut microbiota positively and induce the formation of selected metabolites which indicate the potential prebiotic effects. Intestinal pathology has been linked to changes in gut microbiota composition and function that can affect performance (de Oliveira et al., 2021). As reported by Vigors et al. (2016), changing the absorption mechanism of the small intestine increased nutrient digestibility affecting animal feed efficiency, which is in agreement with what was observed in the current research. The current findings are consistent with Al-Waeli et al. (2021) who stated that RBP supplementation could improve the feed conversion ratio of geese. These authors suggest that the improvement in the feed conversion ratio occurred after the eighth week, with no differences observed in the previous weeks. This fact would explain why previous authors (Kopřiva et al., 2014; Şengül, 2021) did not find differences in this parameter, because they include younger animals in their study.

### Egg quality

No variation was observed among treatments for damaged eggs, egg-breaking strength, eggshell weight, and eggshell thickness. Minerals such as calcium, magnesium, and phosphorus present in the diet can affect eggshell thickness (Khairani and Wiryawan 2016). In the current research, the Ca and P content of the treatment diets was the same for all treatments, and also the eggshell thickness was the same for all experimental groups. Likely, the absence of variations in the mineral content of the diets may explain this fact. Hence, the eggshell-breaking strength and the percentage of damaged eggs were not different among the different experiments. The results of this study on egg external parameters were similar to those reported by Souza et al. (2019) in laying quails. However, a trend was observed for egg-breaking strength, being this parameter higher in quails that had received RBP in the diet than in the control group. Moreover, numerical improvements in eggshell weight were observed when RBP was included in the diets of laying quails as compared with the control, but, these did not achieve a level of statistical significance. Eggshell weight influences eggshell quality (Şekeroǧlu and Altuntaş, 2009). According to Souza et al. (2019), beetroot contains 1.23 mg of manganese, which could increase eggshell weight and improves eggshell quality without any losses in egg production as shown in the current research.

Increasing RBP inclusion from 0 to 0.2% in the diet increased the yolk index. However, adding RBP to the diet up to 0.2% did not cause a significant difference between the yolk of the groups. The results of this study were consistent with those of Şengül (2021) in laying quails. Those authors reported the highest values of the yolk index from the 0.5% group and control group, while the rest of the experimental groups had significantly lower yolk index values. Souza et al. (2019) described a lower value of yolk weight and yolk height when RBP was incorporated into the diet compared to the group that had received corn. However, the inclusion of RBP in the diet showed similar values for yolk weight and height when hens were fed with sorghum. The reason for these differences seems to be unclear and requires further research. The rest of the egg internal parameters (the albumen index and Haugh unit) were unaffected by RBP in the quails’ diet. Ingestion of proteins by laying hens determines the quantity of proteins present in albumen (Souza et al., 2019). As a result, the addition of beetroot to the diets probably did not increase protein, nor did there appear to be a loss in digestion or use of the proteins. Therefore, the albumen index did not differ among the experimental groups. In a similar experiment (Souza et al., 2019), the addition of red beet in diets for laying hens promoted no differences in the albumen weight and albumen height. among the treatments.

Betalains (hydrophilic pigments) are the most researched bioactive compounds in beet. These compounds could contribute to changes in the color of food products (Rey et al., 2005; Kopřiva et al., 2014; de Oliveira et al., 2021). Dietary factors, including the composition of the feeding dosage, are associated with the resultant color (Şengül, 2021). However, a higher concentration (0.8%) of RBP does not affect the color of egg yolk (in terms of lightness, redness, or yellowness). These findings are in line with the observations of Souza et al. (2019), who clarified that the diets with beetroot meal did not change egg yolk color. Similarly, Kopřiva et al. (2014) observed that adding 1 to 2% of beet to laying hen diets did not affect yolk color. Emam and Abdel Wahed (2020) reported that feeding different levels of beet pulp (0 to 15%) not affected yolk color. According to these authors, the absence of changes is possibly due to the hydrophilic chemical nature of betalains, which could interfere with intestinal absorption and subsequent deposition in the yolk.

### TBARS and DPPH concentrations in the yolk

A plant’s antioxidant capacity is mainly derived from phenolic compounds found in its tissues (Souza et al., 2019). There is a high concentration of betanin in beetroot (300–600 mg/kg), which inhibits lipid peroxidation more effectively (Chhikara et al., 2019; Domínguez et al., 2020). The high antioxidant activity of betanin appears to stem from its exceptional electron-donati (Al-Waeli et al., 2021). The antioxidant properties of beetroot prevent DNA, fats, and protein from being damaged by oxidative stress (Chhikara et al., 2019). The TBARS are responsible for determining lipid peroxidation, a critical factor in eggshell durability. Contrary to the expectations, the results obtained in this study showed a linear increase in the TBARS value when RBP was added to the diet (0 to 0.6%). However, the inclusion of 0.8% RBP in the diet provided similar TBARS values to those obtained in the control group. Similar findings were described by Rey et al. (2005) in cooked pork patties. Those authors suggest that higher amounts of red beet could interfere with the determination of the pink TBA chromogen due to the red color of the beetroot extract. Likewise, the DPPH value was reduced when more than 0.6% RBP was incorporated into the diet (*P* < 0.05). Retinol and carotenoids present in red beet are destroyed rapidly by high temperatures. Furthermore, retinol and carotenoids may decrease tocopherol uptake in the gastrointestinal tract, and decreased tocopherol deposition in the yolk may accelerate the oxidative breakdown of fatty acids (Souza et al., 2019). This study was performed during the months of April and June on a local farm in Konya (Türkiye). According to the above, it could be suggested that the high temperature of these months could have interfered with the results obtained in the experiment. Likewise, the improvement of the TBARS value in food products prepared with RBP (Rey et al., 2005) may be due to the direct application of the vegetable. However, the results obtained in this study do not seem clear and require future research. Unfortunately, we have not found any published research on the effects of the inclusion of RBP in the diets of laying quails on the egg yolk TBARS and DPPH value. Most of the previous studies have incorporated RBP as part of the food preparations and not as part of the animal’s diet (Rey et al., 2005; Raikos et al., 2016; Domínguez et al., 2020). Therefore, further research is needed in this field.

Overall, the current findings provide additional support for the use of RBP in the diet of laying quails without affecting their production performance and egg quality. Additionally, considering its positive effects on feed conversion ratio, the addition of 0.4% RBP to laying quail diets may be recommended. by 0.4%. In any case, the results obtained in the current study show that the reuse of may be an acceptable ingredient for laying quails, which could reduce environmental impact and economic losses. Nevertheless, the antioxidant activity of RBP in yolks derived from quail consumption remains unclear and must be explored further.

## Data Availability

The authors declare that all the data and materials used in this study comply with field standards and are available on demand.
